# Supplementary material for: Mapping of promoter usage QTL using RNA-seq data reveals their contributions to complex traits
Source: PLoS Comput Biol. 2022 Aug 29;18(8):e1010436. doi: 10.1371/journal.pcbi.1010436 (PMC9462676; doi:10.1371/journal.pcbi.1010436)
Supplement: S1 Fig — Activity scores of each promoter of the GM12878 cell line are plotted with regression lines. (PDF) [file pcbi.1010436.s001.pdf]

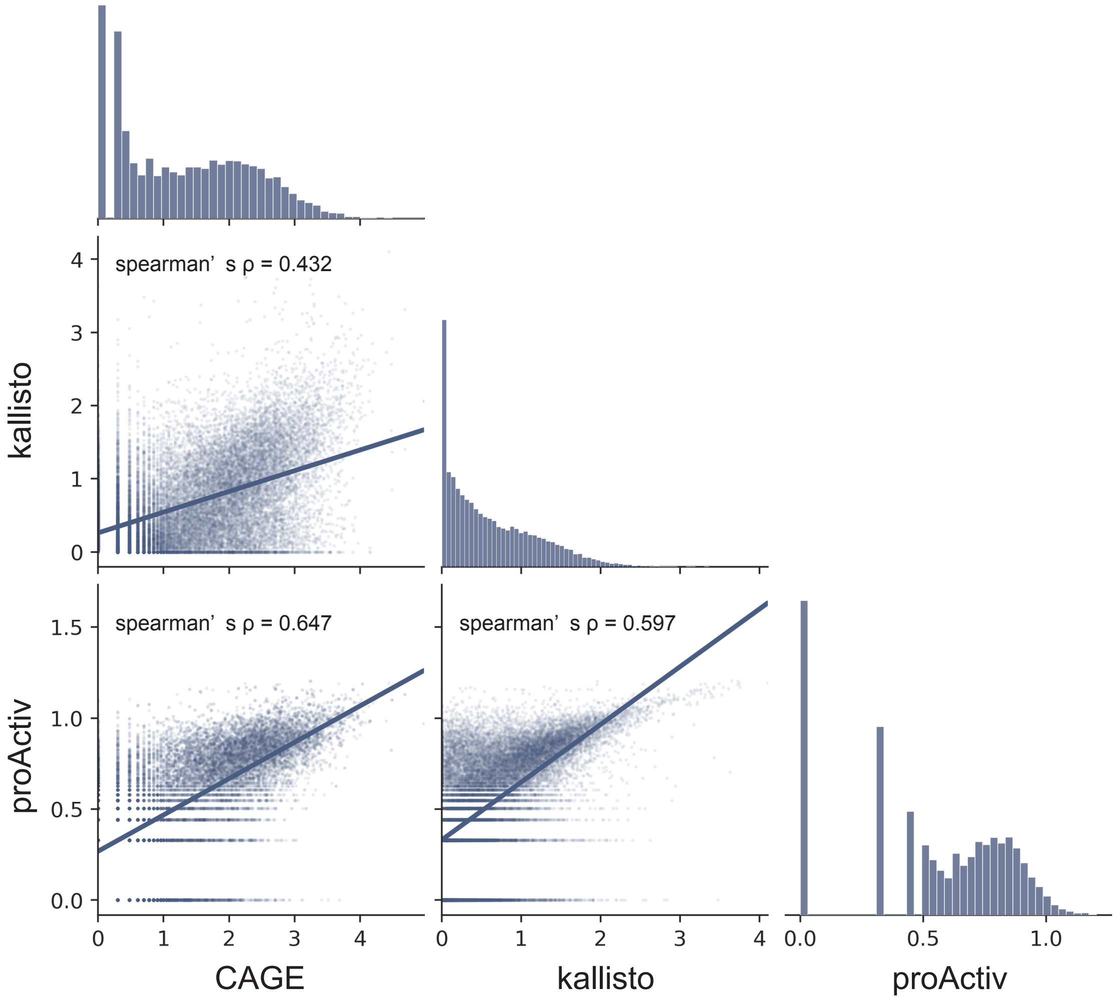

Supplemental Figure 1. Correlation of estimated promoter activites among three different methods. Activity scores of each promoter of the GM12878 cell line are plotted with regression lines.
