# Supplementary material for: Mapping of promoter usage QTL using RNA-seq data reveals their contributions to complex traits
Source: PLoS Comput Biol. 2022 Aug 29;18(8):e1010436. doi: 10.1371/journal.pcbi.1010436 (PMC9462676; doi:10.1371/journal.pcbi.1010436)
Supplement: S2 Fig — 1KGP, the 1000 Genomes Project; MAF, Minor Allele Frequency; GTF, Gene Transfer Format. (PDF) [file pcbi.1010436.s002.pdf]

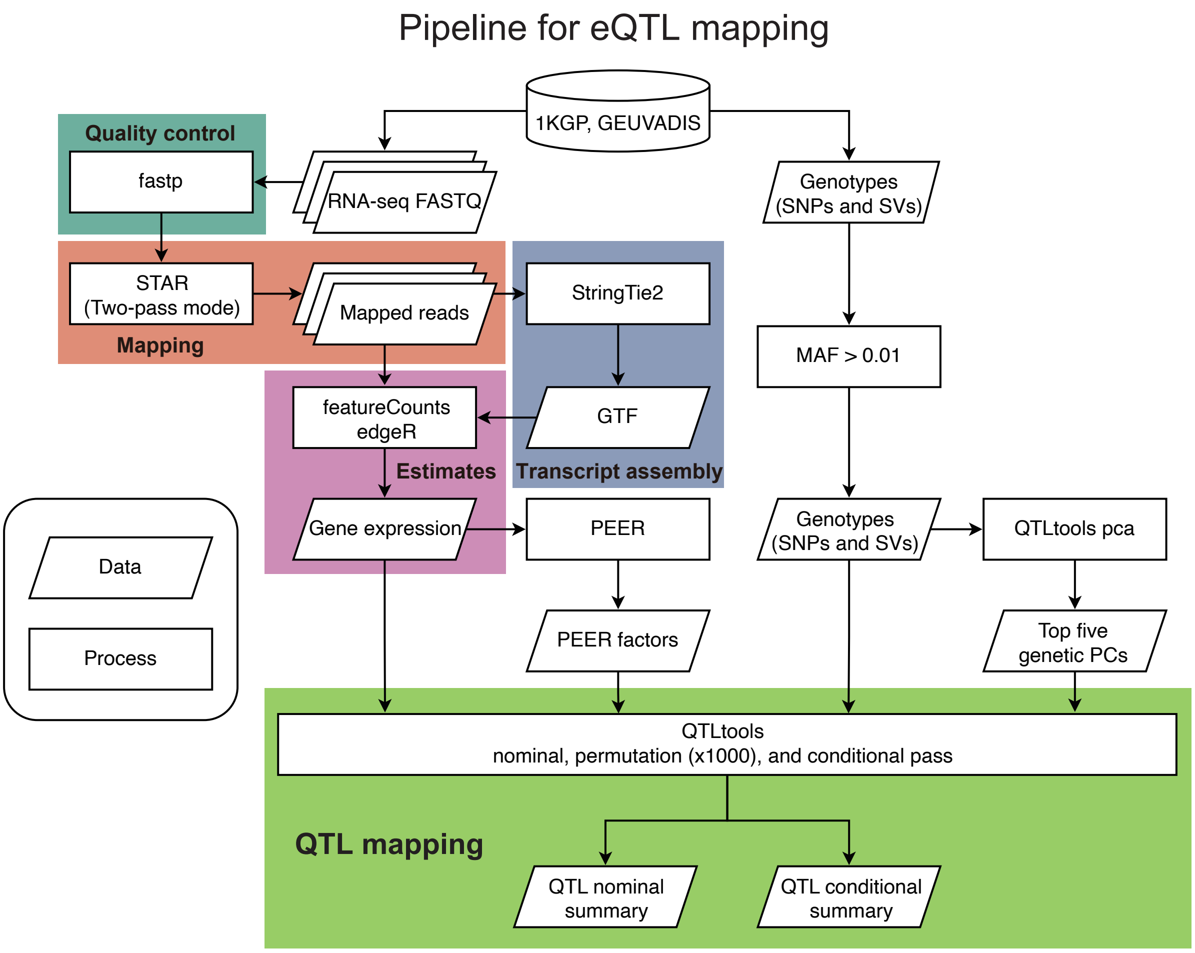

Supplemental Figure 2. Pipeline for eQTL mapping. 1KGP, the 1000 Genomes Project; MAF, Minor Allele Frequency; GTF, Gene Transfer Format.
