# Supplementary material for: Mapping of promoter usage QTL using RNA-seq data reveals their contributions to complex traits
Source: PLoS Comput Biol. 2022 Aug 29;18(8):e1010436. doi: 10.1371/journal.pcbi.1010436 (PMC9462676; doi:10.1371/journal.pcbi.1010436)
Supplement: S3 Fig — (A) Projection on the first two principal components of the normalized genotype data, labelled for populations. GBR, British in England and Scotland; FIN, Finnish in Finland; CEU, Utah residents (CEPH) with Northern and Western European ancestry; YRI, Yoruba in Ibadan, Nigeria; TSI, Toscani in Italia. (B, C) The number of puQTL (B) and eQTL (C) (nominal P < 1.0 × 10^–5^) identified (y-axis) versus the number of PEER factors used (x-axis). Red circles represent the number of PEER factors used for the following analysis. (PDF) [file pcbi.1010436.s003.pdf]

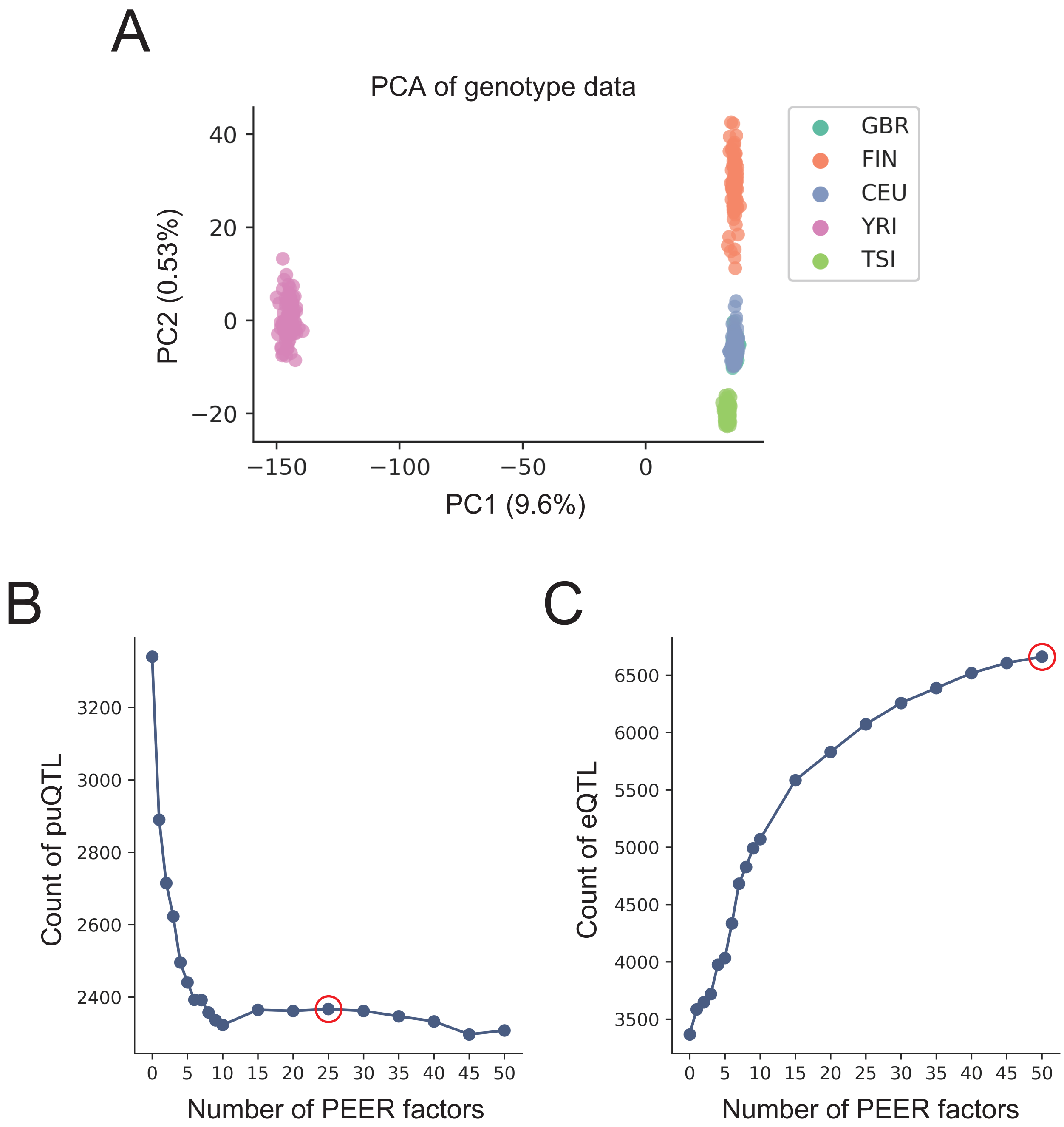

Supplemental Figure 3. Covariates for QTL analysis. (A) Projection on the first two principal components of the normalized genotype data, labelled for populations. GBR, British in England and Scotland; FIN, Finnish in Finland; CEU, Utah residents (CEPH) with Northern and Western European ancestry; YRI, Yoruba in Ibadan, Nigeria; TSI, Toscani in Italia. (B, C) The number of puQTL (B) and eQTL (C) (nominal $P < 1.0 \times 10^{-5}$) identified (y-axis) versus the number of PEER factors used (x-axis). Red circles represent the number of PEER factors used for the following analysis.
