# Supplementary material for: Mapping of promoter usage QTL using RNA-seq data reveals their contributions to complex traits
Source: PLoS Comput Biol. 2022 Aug 29;18(8):e1010436. doi: 10.1371/journal.pcbi.1010436 (PMC9462676; doi:10.1371/journal.pcbi.1010436)
Supplement: S4 Fig — (A) Quantile–quantile plot of P-values. The nominal pass results of chromosome 22 are plotted and a red line indicates expected P-values under the null hypothesis. (B) Distribution of the distance of eQTL best hit variants from the target promoters. (PDF) [file pcbi.1010436.s004.pdf]

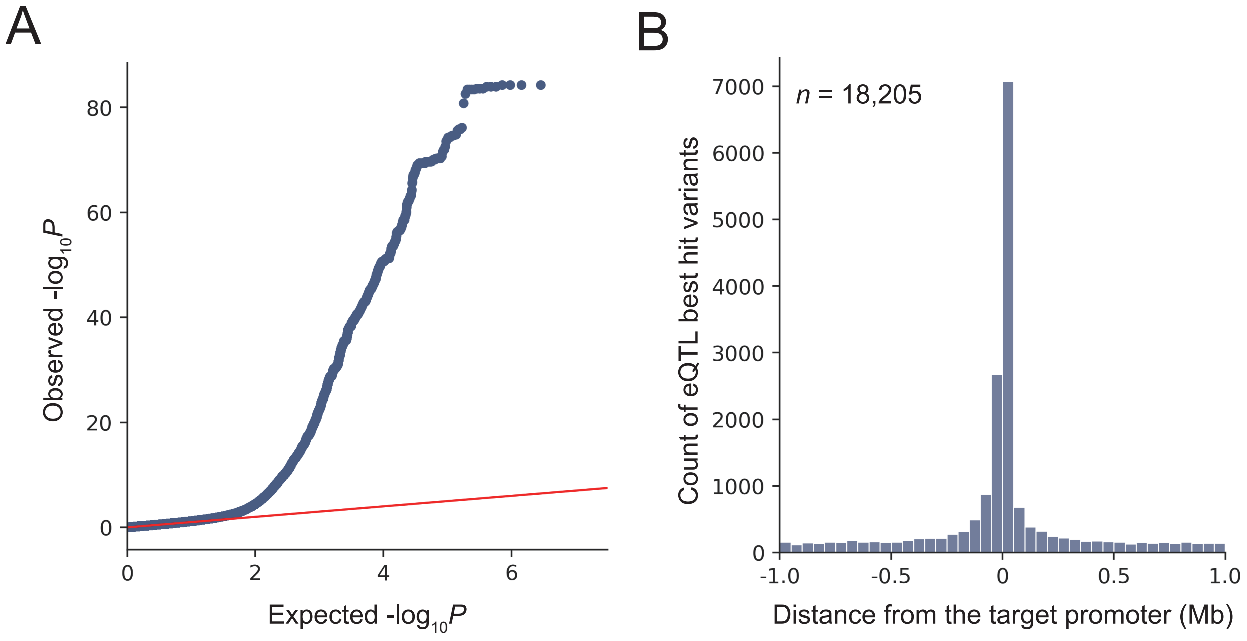

Supplemental Figure 4. eQTL analysis results. (A) Quantile–quantile plot of *P*-values. The nominal pass results of chromosome 22 are plotted and a red line indicates expected *P*-values under the null hypothesis. (B) Distribution of the distance of eQTL best hit variants from the target promoters.
