# Supplementary material for: Mapping of promoter usage QTL using RNA-seq data reveals their contributions to complex traits
Source: PLoS Comput Biol. 2022 Aug 29;18(8):e1010436. doi: 10.1371/journal.pcbi.1010436 (PMC9462676; doi:10.1371/journal.pcbi.1010436)
Supplement: S5 Fig — (A) The TTC23 gene locus. Structures of the TTC23 assembled in this study are shown with ENCODE GM12878 H3K4me3 and H3K27ac ChIP-seq signals. A vertical blue bar indicate the location of an active promoter, prmtr.35339. A black bar indicates the location of a variant rs8028374. (B) Comparison of the promoter activities of the TTC23 gene among rs8028374 genotypes. The numbers in parentheses indicate sample size. (C) The DENND2D gene locus. (D) Comparison of the promoter activities of the DENND2D gene among rs35430374 genotypes. (PDF) [file pcbi.1010436.s005.pdf]

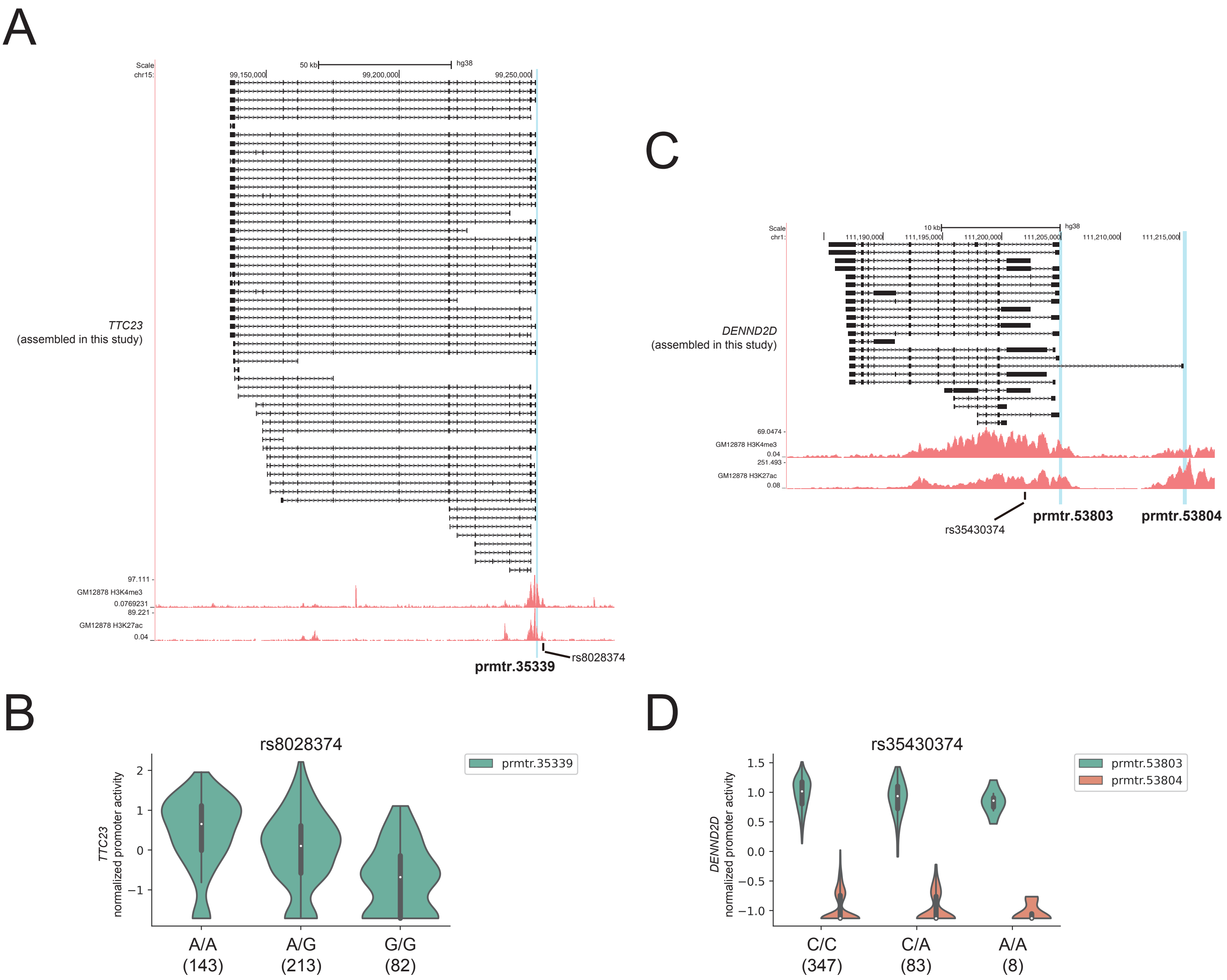

Supplemental Figure 5. Examples of puQTL. (A) The *TTC23* gene locus. Structures of the *TTC23* assembled in this study are shown with ENCODE GM12878 H3K4me3 and H3K27ac ChIP-seq signals. A vertical blue bar indicate the location of an active promoter, prmtr.35339. A black bar indicates the location of a variant rs8028374. (B) Comparison of the promoter activities of the *TTC23* gene among rs8028374 genotypes. The numbers in parentheses indicate sample size. (C) The *DENND2D* gene locus. (D) Comparison of the promoter activities of the *DENND2D* gene among rs35430374 genotypes.
