# Supplementary material for: Mapping of promoter usage QTL using RNA-seq data reveals their contributions to complex traits
Source: PLoS Comput Biol. 2022 Aug 29;18(8):e1010436. doi: 10.1371/journal.pcbi.1010436 (PMC9462676; doi:10.1371/journal.pcbi.1010436)
Supplement: S6 Fig — (A) Propotion of puQTL associations mapped as eQTL. (B) Propotion of puQTL associations mapped as eQTL when expanding linkage disequilibrium (r2 > 0.8). (PDF) [file pcbi.1010436.s006.pdf]

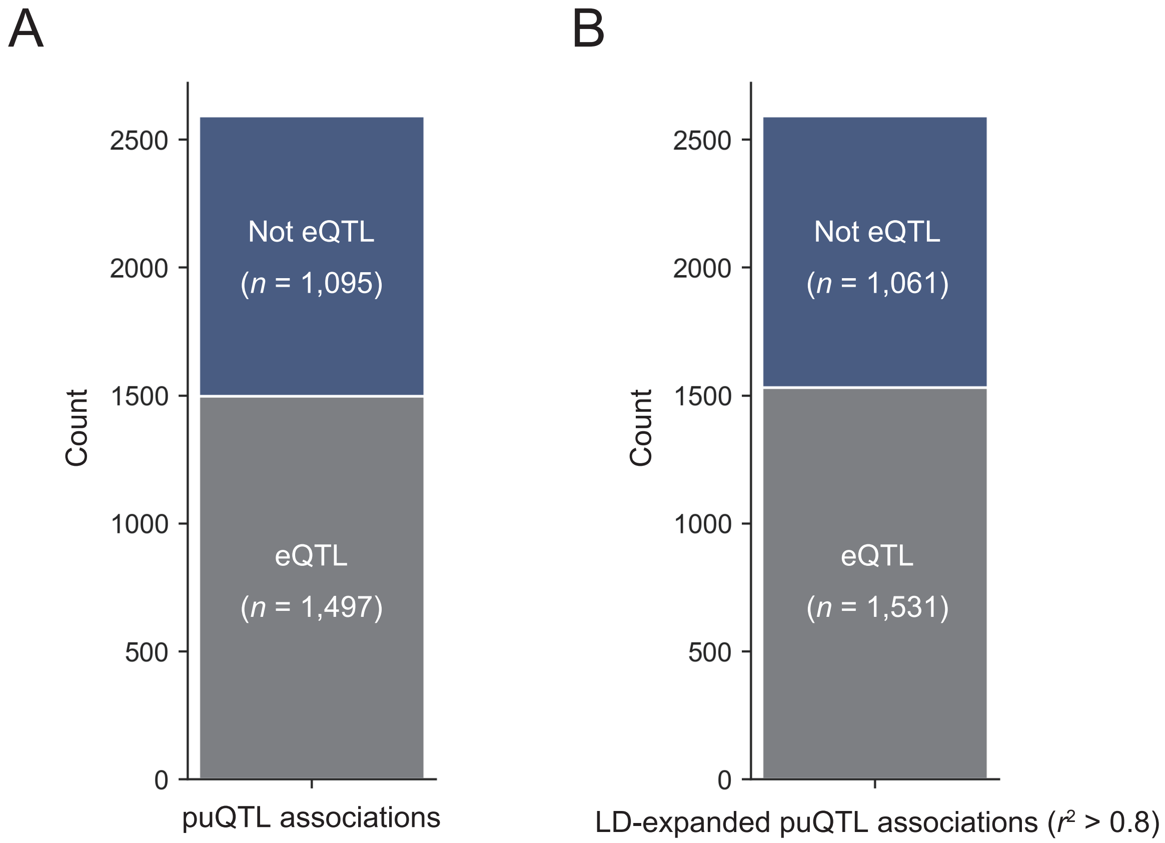

Supplemental Figure 6. Overlaps of puQTL and eQTL associations at the variant level.
(A) Propotion of puQTL associations mapped as eQTL. (B) Propotion of puQTL associations mapped as eQTL when expanding linkage disequilibrium ($r^2 > 0.8$).
