# Supplementary material for: Mapping of promoter usage QTL using RNA-seq data reveals their contributions to complex traits
Source: PLoS Comput Biol. 2022 Aug 29;18(8):e1010436. doi: 10.1371/journal.pcbi.1010436 (PMC9462676; doi:10.1371/journal.pcbi.1010436)
Supplement: S7 Fig — The y-axis represents median values of promoter activity of puQTL genes mapped as eQTL genes (green) and not mapped as eQTL genes (orange) in 438 individuals. (PDF) [file pcbi.1010436.s007.pdf]

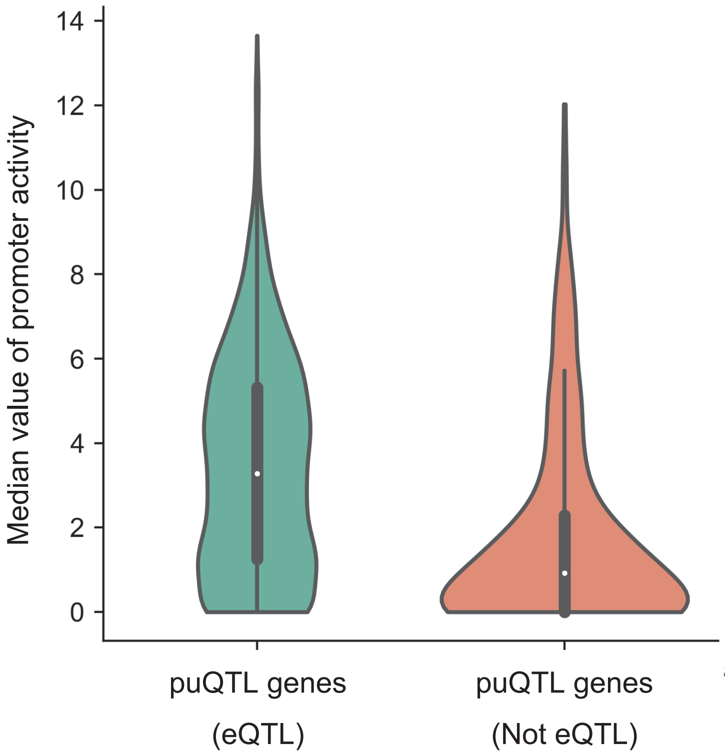

Supplemental Figure 7. Promoter activity of puQTL genes.

The y-axis represents median values of promoter activity of puQTL genes mapped as eQTL genes (green) and not mapped as eQTL genes (orange) in 438 individuals.
