# Supplementary material for: Mapping of promoter usage QTL using RNA-seq data reveals their contributions to complex traits
Source: PLoS Comput Biol. 2022 Aug 29;18(8):e1010436. doi: 10.1371/journal.pcbi.1010436 (PMC9462676; doi:10.1371/journal.pcbi.1010436)
Supplement: S8 Fig — Aggregation plots of histone mark ChIP-seq and ATAC-seq signals for 100 kb regions flanking puQTL best hit variants. Green and blue lines represent aggregated signals for puQTL variants mapped as eQTL and those not mapped as eQTL, respectively. (PDF) [file pcbi.1010436.s008.pdf]

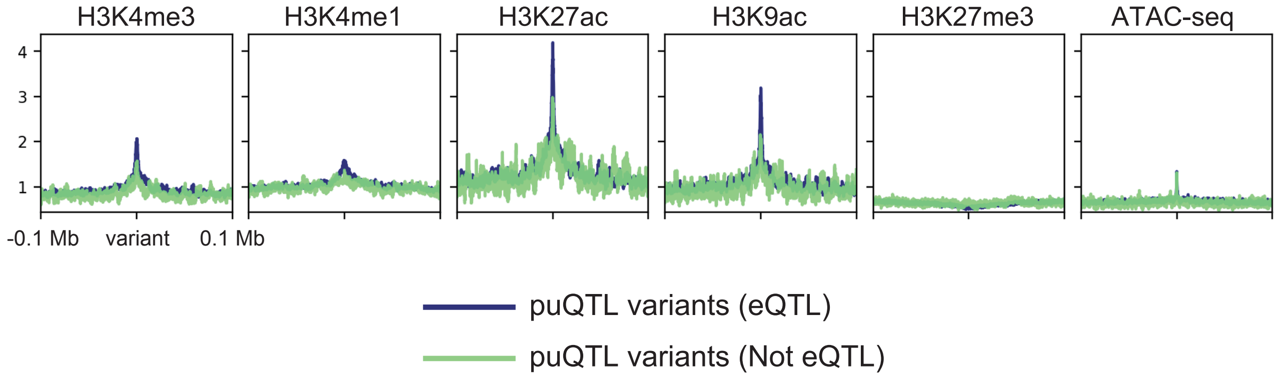

Supplemental Figure 8. Enrichement of epigenetic features of puQTL variants.

Aggregation plots of histone mark ChIP-seq and ATAC-seq signals for 100 kb regions flanking puQTL best hit variants. Blue and green lines represent aggregated signals for puQTL variants mapped as eQTL and those not mapped as eQTL, respectively.
