# Supplementary material for: Mapping of promoter usage QTL using RNA-seq data reveals their contributions to complex traits
Source: PLoS Comput Biol. 2022 Aug 29;18(8):e1010436. doi: 10.1371/journal.pcbi.1010436 (PMC9462676; doi:10.1371/journal.pcbi.1010436)
Supplement: S9 Fig — Enrichment of peaks of histone mark ChIP-seq and transcription factor footprints. Red dots represent significant enrichment at the 5% FDR level and bars show 95% confidence intervals. (PDF) [file pcbi.1010436.s009.pdf]

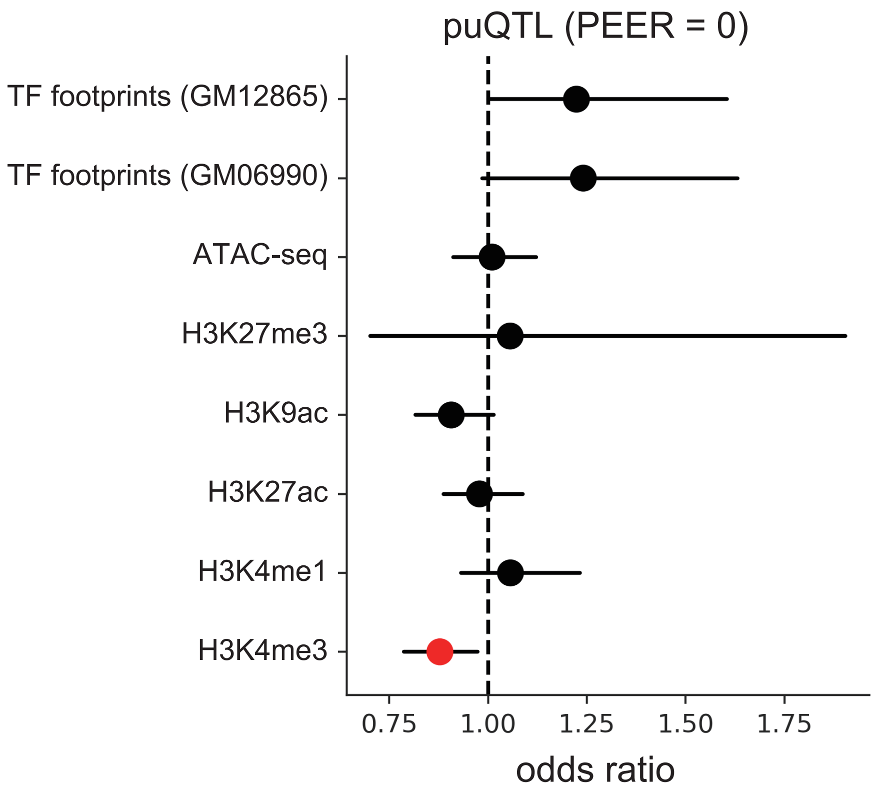

Supplemental Figure 9. Enrichment of puQTL identified without PEER factors in epigenetic features. Enrichment of peaks of histone mark ChIP-seq and transcription factor footprints. Red dots represent significant enrichment at the 5% FDR level and bars show 95% confidence intervals.
