# Supplementary material for: Mapping of promoter usage QTL using RNA-seq data reveals their contributions to complex traits
Source: PLoS Comput Biol. 2022 Aug 29;18(8):e1010436. doi: 10.1371/journal.pcbi.1010436 (PMC9462676; doi:10.1371/journal.pcbi.1010436)
Supplement: S10 Fig — (A) The IVNS1ABP gene locus. Structures of the IVNS1ABP assembled in this study are represented in black with ENCODE GM12878 H3K4me3, H3K4me1, and H3K27ac ChIP-seq signals and H3K27ac HiChIP chromatin interactions. Vertical blue bar indicates the location of an active promoter prmtr.86427. A black bar indicates the location of a structural variant 967_HG00773_ins. (B, C) Comparison of the promoter activities (B) and total expression levels (C) of the IVNS1ABP gene among 967_HG00773_ins genotypes. The numbers in parentheses indicate sample size. (D) Associations of puQTL, fine-mapped puQTL for prmtr.86427, and eQTL for IVNS1ABP are shown in the top, middle, and bottom panel. 967_HG00773_ins is plotted in a red diamond and colors indicate r-squared values between 967_HG00773_ins and other variants. (E) The RSPH1 gene locus. Structures of the RSPH1 in the Ensembl 104 annotation and assembled in this study are represented in black and red, respectively with ENCODE GM12878 H3K4me3 and H3K27ac ChIP-seq signals. Vertical blue bars indicate the location of an active promoters. A black bar indicates the location of a structural variant 28764_HG02059_ins. (F, G) Comparison of the promoter activities (F) and total expression levels (G) of the RSPH1 gene among 28764_HG02059_ins genotypes. (H) Associations of puQTL, fine-mapped puQTL for prmtr.70068, and eQTL for RSPH1 are shown in the top, middle, and bottom panel. 28764_HG02059_ins is plotted in a red diamond and colors indicate r-squared values between 28764_HG02059_ins and other variants. (PDF) [file pcbi.1010436.s010.pdf]

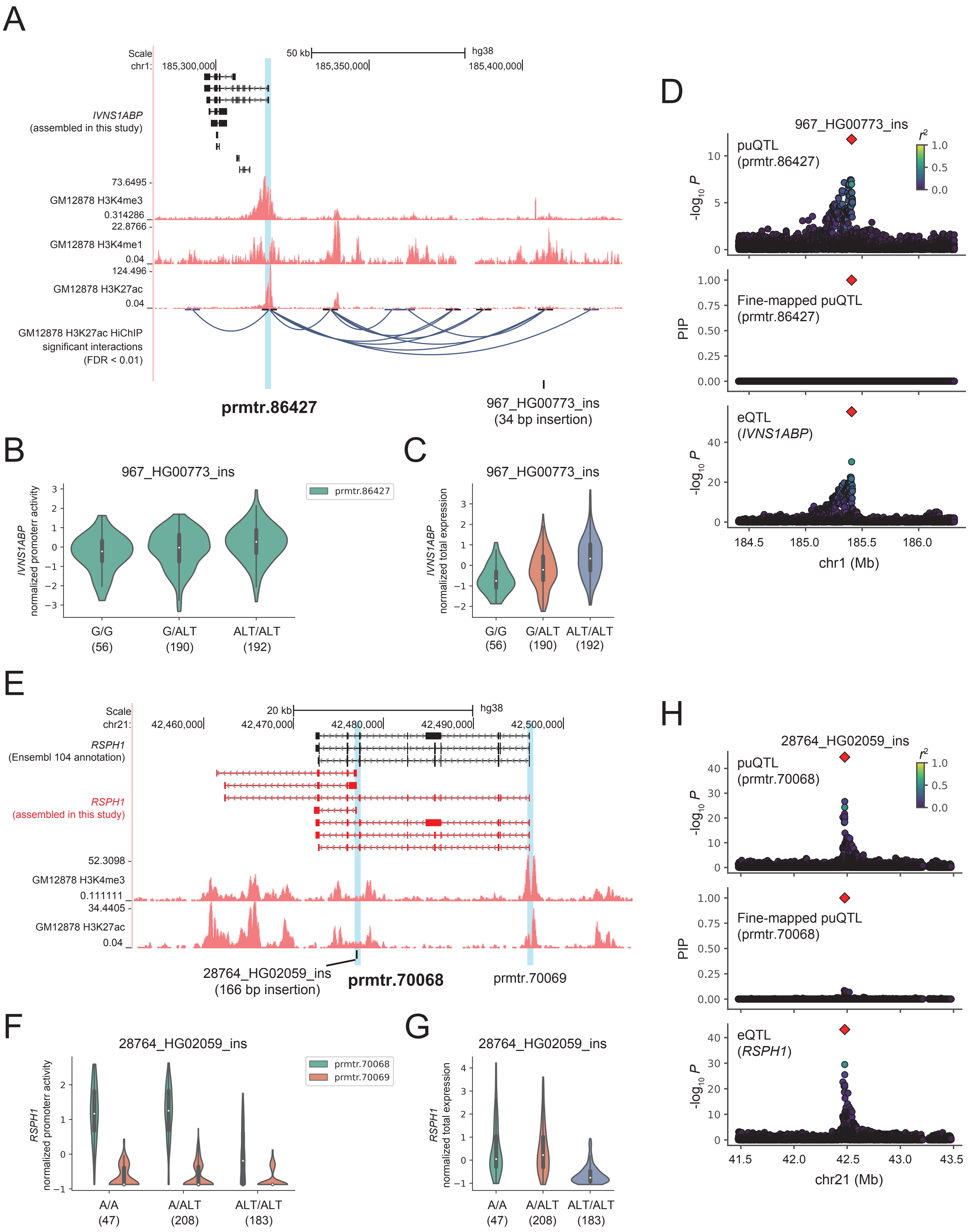

A
Scale
chr1:
50 kb
hg38
185,300,000
185,350,000
185,400,000
IVNS1ABP
(assembled in this study)
73.6495
GM12878 H3K4me3
0.314286
22.8766
GM12878 H3K4me1
0.04
124.496
GM12878 H3K27ac
0.04
GM12878 H3K27ac HiChIP
significant interactions
(FDR < 0.01)
prmtr.86427
967_HG00773_ins
(34 bp insertion)
B
967_HG00773_ins
prmtr.86427
IVNS1ABP
normalized promoterr activity
G/G
(56)
G/ALT
(190)
ALT/ALT
(192)
C
967_HG00773_ins
IVNS1ABP
normalized total expression
G/G
(56)
G/ALT
(190)
ALT/ALT
(192)
D
967_HG00773_ins
puQTL
(prmtr.86427)
-log10 P
r2
Fine-mapped puQTL
(prmtr.86427)
PIP
eQTL
(IVNS1ABP)
184.5
185.0
185.5
186.0
chr1 (Mb)
E
Scale
chr21:
20 kb
hg38
42,460,000
42,470,000
42,480,000
42,490,000
42,500,000
RSPH1
(Ensembl 104 annotation)
RSPH1
(assembled in this study)
52.3098
GM12878 H3K4me3
0.111111
34.4405
GM12878 H3K27ac
0.04
28764_HG02059_ins
(166 bp insertion)
prmtr.70068
prmtr.70069
F
28764_HG02059_ins
prmtr.70068
prmtr.70069
RSPH1
normalized promoterr activity
A/A
(47)
A/ALT
(208)
ALT/ALT
(183)
G
28764_HG02059_ins
RSPH1
normalized total expression
A/A
(47)
A/ALT
(208)
ALT/ALT
(183)
H
28764_HG02059_ins
puQTL
(prmtr.70068)
-log10 P
r2
Fine-mapped puQTL
(prmtr.70068)
PIP
eQTL
(RSPH1)
41.5
42.0
42.5
43.0
43.5
chr21 (Mb)

Supplemental Figure 10. Structural variants associated with promoter usage. (A) The *IVNS1ABP* gene locus. Structures of the *IVNS1ABP* assembled in this study are represented in black with ENCODE GM12878 H3K4me3, H3K4me1, and H3K27ac ChIP-seq signals and H3K27ac HiChIP chromatin interactions. Vertical blue bar indicates the location of an active promoter prmtr.86427. A black bar indicates the location of a structural variant 967_HG00773_ins. (B, C) Comparison of the promoter activities (B) and total expression levels (C) of the *IVNS1ABP* gene among 967_HG00773_ins genotypes. The numbers in parentheses indicate sample size. (D) Associations of puQTL, fine-mapped puQTL for prmtr.86427, and eQTL for *IVNS1ABP* are shown in the top, middle, and bottom panel. 967_HG00773_ins is plotted in a red diamond and colors indicate r-squared values between 967_HG00773_ins and other variants. (E) The *RSPH1* gene locus. Structures of the *RSPH1* in the Ensembl 104 annotation and assembled in this study are represented in black and red, respectively with ENCODE GM12878 H3K4me3 and H3K27ac ChIP-seq signals. Vertical blue bars indicate the location of an active promoters. A black bar indicates the location of a structural variant 28764_HG02059_ins. (F, G) Comparison of the promoter activities (F) and total expression levels (G) of the *RSPH1* gene among 28764_HG02059_ins genotypes. (H) Associations of puQTL, fine-mapped puQTL for prmtr.70068, and eQTL for *RSPH1* are shown in the top, middle, and bottom panel. 28764_HG02059_ins is plotted in a red diamond and colors indicate r-squared values between 28764_HG02059_ins and other variants.
