# Supplementary material for: Mapping of promoter usage QTL using RNA-seq data reveals their contributions to complex traits
Source: PLoS Comput Biol. 2022 Aug 29;18(8):e1010436. doi: 10.1371/journal.pcbi.1010436 (PMC9462676; doi:10.1371/journal.pcbi.1010436)
Supplement: S11 Fig — (A) The MUC12-AS1 gene locus. Structures of the MUC12-AS1 in the Ensembl 104 annotation and assembled in this study are represented in black and red, respectively, with ENCODE GM12878 H3K4me3 and H3K27ac ChIP-seq signals. Vertical blue bars indicate the location of active promoters. A black bar indicates the location of a variant rs10229453. (B, C) Comparison of the promoter activities (B) and total expression levels (C) of the MUC12-AS1 gene among rs10229453 genotypes. The numbers in parentheses indicate sample size. (D) Associations of puQTL, fine-mapped puQTL for prmtr.99696, and eQTL for MUC12-AS1 are shown in the top, middle, and bottom panel. rs10229453 is plotted in a red diamond and colors indicate r-squared values between rs10229453 and other variants. (PDF) [file pcbi.1010436.s011.pdf]

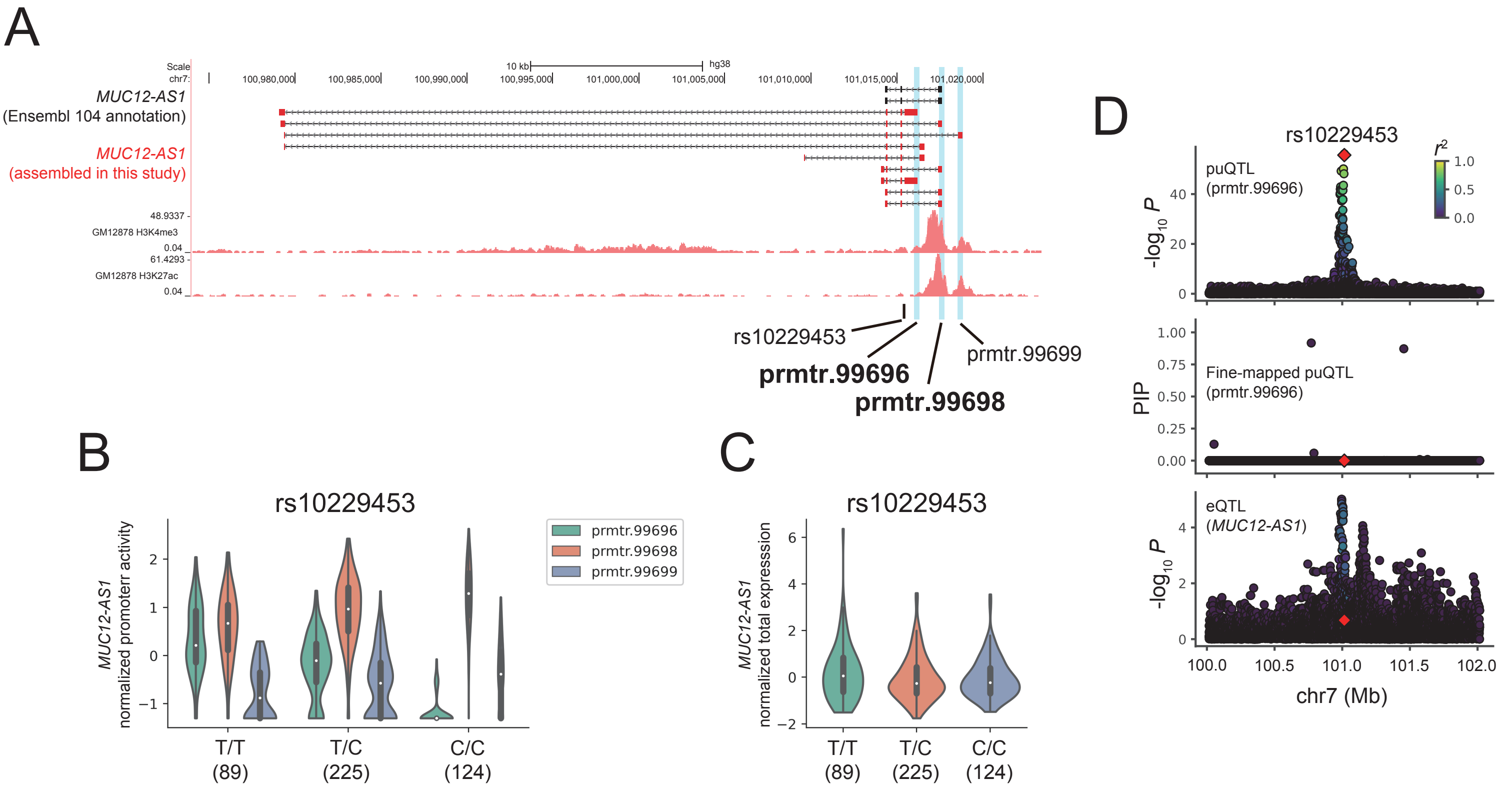

Supplemental Figure 11. puQTL associated with distinct promoters of the *MUC12-AS1* gene with opposite effects. (A) The *MUC12-AS1* gene locus. Structures of the *MUC12-AS1* in the Ensembl 104 annotation and assembled in this study are represented in black and red, respectively, with ENCODE GM12878 H3K4me3 and H3K27ac ChIP-seq signals. Vertical blue bars indicate the location of active promoters. A black bar indicates the location of a variant rs10229453. (B, C) Comparison of the promoter activities (B) and total expression levels (C) of the *MUC12-AS1* gene among rs10229453 genotypes. The numbers in parentheses indicate sample size. (D) Associations of puQTL, fine-mapped puQTL for prmtr.99696, and eQTL for *MUC12-AS1* are shown in the top, middle, and bottom panel. rs10229453 is plotted in a red diamond and colors indicate r-squared values between rs10229453 and other variants.
