# Supplementary material for: Mapping of promoter usage QTL using RNA-seq data reveals their contributions to complex traits
Source: PLoS Comput Biol. 2022 Aug 29;18(8):e1010436. doi: 10.1371/journal.pcbi.1010436 (PMC9462676; doi:10.1371/journal.pcbi.1010436)
Supplement: S12 Fig — (A) Multiple sequence alignment of two isoforms of the Sin1 (MAPKAP1) protein. Protein sequences of full-length Sin1 (SIN1_HUMAN) and NTD-lacking Sin1 (BIAMA5_HUMAN) are aligned in top and bottom rows, respectively. Asterisks (*) indicate positions of matched residues. Red, black, and blue rectangles indicate sequences of N-terminal domain, conserved region in middle domain, and Preckstrin-homology domain, respectively. (B, C) Protein structures of full-length Sin1 (SIN1_HUMAN) (B) and NTD-lacking Sin1 (BIAMA5_HUMAN) (C) predicted by AlphaFold2 with colors representing per-residue confidence score. Dotted circles indicate N-terminal domain. (PDF) [file pcbi.1010436.s012.pdf]

A

```
CLUSTAL multiple sequence alignment by MUSCLE (3.8)

sp|Q9BPZ7-2|SIN1_HUMAN      MAFLDNPTIILAHIRQSHVTSDDTGMCEMVLIDHDVDLEKIHPPSMPGDSGSEIQGSNGE
tr|B1AMA5|B1AMA5_HUMAN      ------------------------------------------------------------

sp|Q9BPZ7-2|SIN1_HUMAN      TQGYVYAQSVDITSSWDFGIRRRSNTAQRLERLRKERQNQIKCKNIQWKERNSKQSAQEL
tr|B1AMA5|B1AMA5_HUMAN      ------------------------------------------------------------

sp|Q9BPZ7-2|SIN1_HUMAN      KSLFEKKSLKEKPPISGKQSILSVRLEQCPLQLNNPFNEYSKFDGKGHVGTTATKKIDVY
tr|B1AMA5|B1AMA5_HUMAN      ------------------------------------------------------------

sp|Q9BPZ7-2|SIN1_HUMAN      LPLHSSQDRLLPMTVVTMASARVQDLIGLICWQYTSEGREPKLNDNVSAYCLHIAEDDGE
tr|B1AMA5|B1AMA5_HUMAN      ------------MTVVTMASARVQDLIGLICWQYTSEGREPKLNDNVSAYCLHIAEDDGE
                                        ************************************************

sp|Q9BPZ7-2|SIN1_HUMAN      VDTDFPPLDSNEPIHKFGFSTLALVEKYSSPGLTSKESLFVRINAAHGFSLIQVDNTKVT
tr|B1AMA5|B1AMA5_HUMAN      VDTDFPPLDSNEPIHKFGFSTLALVEKYSSPGLTSKESLFVRINAAHGFSLIQVDNTKVT
                            ************************************************************

sp|Q9BPZ7-2|SIN1_HUMAN      MKEILLKAVKRRKGSQKVSGSRADGVFEEDSQIDIATVQDMLSSHHYKSFKVSMIHRLRF
tr|B1AMA5|B1AMA5_HUMAN      MKEILLKAVKRRKGSQKVSGSRADGVFEEDSQIDIATVQDMLSSHHYKSFKVSMIHRLRF
                            ************************************************************

sp|Q9BPZ7-2|SIN1_HUMAN      TTDVQLGISGDKVEIDPVTNQKASTKFWIKQKPISIDSDLLCACDLAEEKSPSHAIFKLT
tr|B1AMA5|B1AMA5_HUMAN      TTDVQLGISGDKVEIDPVTNQKASTKFWIKQKPISIDSDLLCACDLAEEKSPSHAIFKLT
                            ************************************************************

sp|Q9BPZ7-2|SIN1_HUMAN      YLSNHDYKHLYFESDAATVNEIVLKVNYILESRASTARADYFAQKQRKLNRRTSFSFQKE
tr|B1AMA5|B1AMA5_HUMAN      YLSNHDYKHLYFESDAATVNEIVLK-----------------------------------
                            *************************

sp|Q9BPZ7-2|SIN1_HUMAN      KKSGQQ
tr|B1AMA5|B1AMA5_HUMAN      ------
```

[red box] : N-terminal domain
[black box] : Conserved region in middle domain
[blue box] : Preckstrin-homology domain

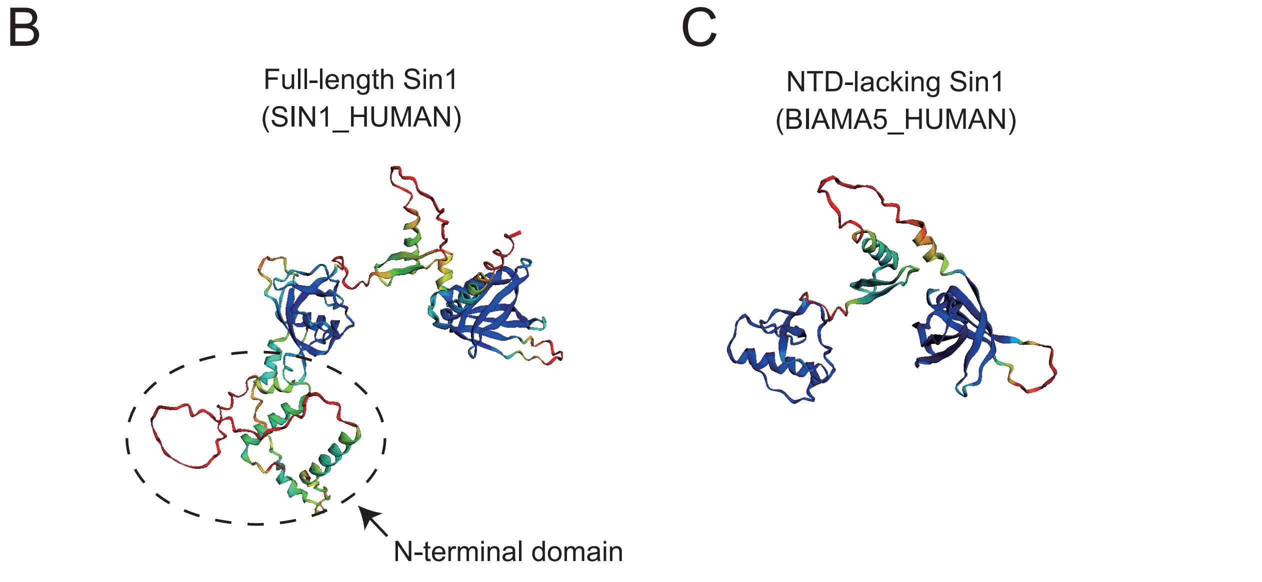

Supplemental Figure 12. Comparison of the Sin1 proteins translated from distinct transcripts. (A) Multiple sequence alignment of two isoforms of the Sin1 (MAPKAP1) protein. Protein sequences of full-length Sin1 (SIN1_HUMAN) and NTD-lacking Sin1 (BIAMA5_HUMAN) are alingned in top and bottom rows, respectively. Asterisks (*) indicate positions of matched residues. Red, black, and blue rectangles indicate sequences of N-terminal domain, conserved region in middle domain, and Preckstrin-homology domain, respectively. (B, C) Protein structures of full-length Sin1 (SIN1_HUMAN) (B) and NTD-lacking Sin1 (BIAMA5_HUMAN) (C) predicted by AlphaFold2 with colors representing per-residue confidence score. Dotted circles indicate N-terminal domain.
