# Supplementary material for: Mapping of promoter usage QTL using RNA-seq data reveals their contributions to complex traits
Source: PLoS Comput Biol. 2022 Aug 29;18(8):e1010436. doi: 10.1371/journal.pcbi.1010436 (PMC9462676; doi:10.1371/journal.pcbi.1010436)
Supplement: Attachment — Submitted filename: RebuttalLetter_20220701.docx [file pcbi.1010436.s014.docx]

Dear Managing Editors,

Thank you very much for handling our manuscript entitled “Mapping of promoter usage QTL using RNA-seq data reveals their contributions to complex traits” (PCOMPBIOL-D-22-00463). We are also grateful to the referees for their contributions and acknowledge their constructive comments. We have rewritten the manuscript according to the comments.

You can find below our answers to the referees. We believe we have addressed all the points raised by the referees and would like to submit this revised version for possible publication.

Yours Sincerely,

Mikita Suyama

–

Reviewer #1: Kubota and Suyama present a manuscript describing a new method for the identification of associations between the presence of genetic variants and the activity of promoters using RNA-Seq data. This method allows more precision during the functional interpretation of GWAS variants, as well as to identify some signals that traditional eQTL cannot detect with its bulk-based approach to quantify gene expression. The authors assess the value of this method, puQTL, using RNA-Seq and variation from cancer cell lines, comparing the results with CAGE-based methods, and describe supporting facts and cases at functional level. In my opinion, this is an interesting and promising method to start advancing in the functional interpretation of GWAS variants, and to start overcoming common simplifications and limitations of current eQTL analyses. The manuscript is well written and clear.

I have though some concerns, mostly regarding the evaluation of the method and its components:

- In page 9, the authors claim that proActive estimations of promoter activity were “correlated more strongly” with CAGE, than the kallisto method. But they do not describe the level and type of correlation. Even assuming the limitations of the CAGE method, it is necessary to better describe the scope of proActive, as it is essential to understand which promoters are, or are not considered inside puQTL.

We thank the reviewer for pointing this out. We assessed the accuracy of promoter activity estimates focusing on spearman’s ρ between those estimated by CAGE, proActiv, and kallisto (page 9, line number 246-248, Figure S1). We found that promoter activities estimated by CAGE are correlated more strongly with those estimated by proActiv (spearman’s ρ = 0.647) than those estimated by kallisto (spearman’s ρ = 0.432), thus concluded that proActiv is more suitable to our puQTL study.

The scope of proActiv is all promoters except for those of single-exon genes and those that overlapped with internal exons, which are not uniquely identifiable from splice junctions, because proActiv leverages junction reads mapped on the first and second exon to estimate promoter activities. We calculated spearman’s ρ described above using the all analyzable promoters.

- I think it is important to understand better the nature of the 16.1% of puQTL, not captured by eQTL, as it constitutes the main benefit of this method. Around this comparison, there are several questions that need to be answered and also discussed. For example:

- is the puQTL vs eQTL signals comparison based on variant, or on LD-block?.

We thank the reviewer for the question. The comparison between puQTL vs. eQTL signals described in the manuscript is based on “genes”.

- How many associations match at variant level and at LD level?

We tested how many best hit puQTL variants or LD-expanded (*r*^2^ > 0.8) puQTL variants are mapped as eQTL. We found that 57.8% puQTL associations (1,497/2,592) match with eQTL associations at variant level, and 59.1% puQTL associations (1,531/2,592) match with eQTL associations at LD level (*r*^2^ > 0.8) (Figure S6). The difference in gene level matching and variant level matching indicates that a proportion of puQTL genes are associated with distinct loci from eQTL genes. We added the sentences on page 11, line number 293-294.

- Is the absence of promoter definition by proActive, the reason for the different number of overall signals (2592 vs 18205)?

We thank the reviewer for the question. One reason for the difference in total number of signals between puQTL and eQTL is probably the limited power to estimate promoter activity. While all reads mapped to exons are used to quantify gene expression levels (eQTL), only junction reads spanning the first and second exons are used to estimate promoter activity (puQTL). This reduces the number of reads that can be used to quantify, reducing the accuracy of the promoter activity estimation and the power of mapping QTL. We believe this is one of the major reasons why the total number of signals differs between puQTL and eQTL. We added the sentences on page 10, line number 274-279.

- What is the number of input genes/expression considered for puQTL and eQTL?

The numbers of input phenotypes for puQTL (promoters) and eQTL (genes) mapping are 12,957 and 12,275, respectively. We added the sentences on page 10, line number 267-268.

- Does the 16.1% correlate more or less with epigenetics signals, than the other 83.9% puQTL overlapping eQTLs? As for chromatin non-associated puQTLs, is this 16,1% of new signals also more enriched in non-PEER?

We thank the reviewer for the valuable comment. We checked epigenetic signals around unique and eQTL-overlapping puQTL variants, and found that unique puQTL variants show relatively weaker enrichment of active epigenetic marks (H3K4me3 and H3K27ac) than eQTL-overlapping puQTL variants (Figure S8). This result indicates a possibility that unique puQTL variants tend to be located in lowly activated regulatory regions, or include false positive signals probably caused by limited power for the detection of lowly-exprepressed promoters. We added the sentences on page 12, line number 319-323.

In non-PEER mapping, we could not detect the same puQTL signals detected by 25 PEER mapping, thus we cannot answer to the latter comment.

- As one would expect, is the 16% of puQTL unique signals covering mostly multi-promoter genes? If there are single-promoter genes within this 16%, any idea of why they were not detected by eQTL?

We performed puQTL-associated gene classification for 310 puQTL genes not overlapping eQTL genes as well as Figure 3A-B. We found that 76.5% puQTL genes (237/310) harbor multiple promoters and 23.5% puQTL genes (73/310) harbor single promoters, indicating that puQTL genes identified in study might include false positive signals. As we mentioned above, only junction reads spanning the first and second exons are used to estimate promoter activity, reducing the accuracy of the promoter activity estimation and the power of mapping QTL. That is a limitation of this study but we believe that our pipeline is robust when mapping QTL associated with moderately or highly expressed promoters, as we showed in some examples in the manuscript (Figure 3, 4). We now explicitly acknowledge this limitation of our analysis in the Results and Discussion sections (page 13, line number 348-353 and page 18, line number 483-485).

- As for the TMEM200A example, what is the overall limitation expected from the Ensemble file, and how this could be improved. Could we improve sensitivity by being more inclusive when predicting isoforms? Please discuss.

We thank the reviewer for the valuable comment. As we pointed out in the manuscript, the Ensembl annotation of human genes is incomplete. It is because promoters expressed in a small proportion of the human population, which may be regulated by genomic variations, are likely to be overlooked when annotation files are constructed based on limited RNA-seq data. However, some major promoters, like the *TMEM200A* example, somehow have been missing. We are not exactly sure why some highly expressed promoters are not described in the Ensembl annotation file (probably this reflects limitations of the Ensembl pipeline and manual curation by humans), but in general, we believe that we can improve sensitivity of isoform prediction by incorporating mapped reads from as many samples as possible. We added the sentences on page 20, line number 527-533.

- In my opinion, the authors should emphasize in the discussion (and backup with numbers in results) the added value of this methodology, beyond single representative cases. For example, how many new associations should we expect in the case we would apply this method to GTEX? What are the kinds of genes where we expect a gain? Are the associations with multipromoter genes expected to co-localize more with quantitative traits, vs binary traits?

We thank the reviewer for the suggestion. In this study, we mapped 2,592 puQTL associations and 1,927 puQTL genes using 438 samples. As for GTEx tissues with >100 sample size, assuming that the power of puQTL mapping increases with increasing sample size, we expect that 674 ~ 4,177 puQTL associations and 501 ~ 3,106 puQTL genes per tissue would be mapped by our pipeline. Assuming that new associations would be identified at the same ratio with this study (16.1%), we expect that 80 ~ 500 genes per tissue would be identified as new associations. We added the sentences on page 18, line number 489-494.

As well as eQTL associations, however, it would be difficult to estimate what kinds of puQTL genes are newly mapped and what type traits tend to co-localize with puQTL.

Minor:

Page 9, “Ensemble gene annotation file” is vague. Which type of genes are considered? What is in the GTF file? Coding and not coding? This is not clear from the main text.

We thank the reviewer for pointing this out. “Ensembl gene annotation file” is a GTF file describing the structure of all genes, including coding and non-coding. We downloaded this file from <http://ftp.ensembl.org/pub/release-104/gtf/homo_sapiens/Homo_sapiens.GRCh38.104.gtf.gz> . We added the sentences on page 10, line number 262-263.

Pge 10, please further clarify the example described for rs802…. and rs354… . What do the authors try to demonstrate? The absence of intragenic signals? Now, it is difficult to understand.

We thank the reviewer for the comment. We show that rs8028374 was mapped as puQTL associated with the most external promoters of the *TTC23* gene, as the previous CAGE-based puQTL study (Garieri et al., 2017) has shown. As for the *DENND2D* gene, rs35430374 was not mapped as puQTL associated with the external promoters, in concordance with the result of the previous study. Overall, in Figure S5 and on page 11, line number 282-289, we would like to show that our results of puQTL mapping are consistent with the results of the previous puQTL analysis.

Reviewer #2: This study by Kubota and Suyama seeks to map QTLs associated with variation in promoter usage in the GEUVADIS population dataset. This is a very important undertaking to understand the mechanisms of gene expression and map genetic variants that directly regulate variation in transcription initiation. In general, the study is fairly comprehensive and well written. However, I have a few major concerns to be addressed:

(1) It is unclear to me that this manuscript presents a unified computational framework with which to identify puQTL. While the study uses a different software to quantify promoter usage, this software itself has been published elsewhere, and the rest of the pipeline involves a standard set of bioinformatic QC, filtering, and data cleaning up steps that are not novel in and of themselves. The authors should make it more clear why they refer to this as a new framework (to me, indicating an algorithm, set of algorithms, or innovative way of thinking of or implementing a computational problem or pipeline)

We thank the reviewer for the valuable comment. Indeed, as you point out, we may have misdefined the term “computational framework”. A word such as “a new method” would be more appropriate for this study. We now consistently use "a new method of puQTL mapping" in the manuscript.

(2) I am still a bit unclear which molecular metric or phenotype is being mapping. Specifically, are the authors mapping isoforms levels (by TPMs), differential promoter usage (ratios of promoter usage within a gene), or some other metric? The choice of metric drastically affects the interpretation of the QTLs and the expected overlap with eQTLs, etc. For instance, the authors state that 212 puQTL genes have a single promoter – are all of these puQTL then also eQTL (since presumably this is mapping isoform levels)?

We thank the reviewer for the question. The metric used for our puQTL mapping is absolute (not relative) promoter activity estimated by proActiv. proActiv performs normalization of the number of junction reads spanning the first and second exons using DESeq2, which is well used software for normalization of gene expression, and log2 transformation to obtain absolute promoter activity.

We found that 65.6% puQTL genes having a single promoter (139/212) are also eQTL genes, but 34.4% (73/212) genes are not. This is probably due to the limited power to estimate promoter activity. While all reads mapped to exons are used to quantify gene expression levels (eQTL), only junction reads spanning the first and second exons are used to estimate promoter activity (puQTL). This reduces the number of reads that can be used to quantify, reducing the accuracy of the promoter activity estimation and leading to false positive puQTL signals. We believe this is one of the major reasons why not all puQTL genes harboring single promoters are eQTL genes. We now explicitly acknowledge this limitation of our analysis in the Results and Discussion sections (page 11, line number 299-301; page 13, line number 342-343; page 18, line number 483-485).

(3) The authors state that 16.1% of genes associated with puQTLs did not overlap with those associated with eQTLs. It would be nice to perform further analyses on characteristics of these genes to identify either technical or biological features that differentiate these genes. Do they tend to have multiple promoters whose usage balances out to maintain constant gene expression levels? Is there an bias towards lower or higher expressed genes in this group and thus either QTL methods is underpowered to identify QTL?

We thank the reviewer for the valuable comment. We checked epigenetic signals around 16.1% and other 83.9% puQTL variants, and found that unique puQTL variants (16.1%) show relatively weaker enrichment of active epigenetic marks (H3K4me3 and H3K27ac) than eQTL-overlapping puQTL variants (Figure S8). This result indicates a possibility that unique puQTL variants tend to be located in lowly activated regulatory regions, or include false positive signals probably caused by limited power for the detection of lowly-expressed promoters. We added the sentences on page 12, line number 319-323.

We performed puQTL-associated gene classification for 310 puQTL genes not overlapping eQTL genes as well as Figure 3A-B. We found that 237 puQTL genes harbor multiple promoters and 225 puQTL genes have only one promoter associated with puQTL, while only 12 genes have multi-promoters associated with puQTL. This result indicates that a possible reason why 16.1% puQTL genes did not overlap with eQTL genes is that the genes have lowly-expressed puQTL-associated promoters that do not account for a large proportion of total gene expression. We added the sentences on page 13, line number 348-353.

In addition, we found that 16.1% of puQTL genes not overlapping with eQTL genes have lower promoter activities than those overlapping with eQTL genes (Figure S7). This bias toward lower promoter activities might lead to limited power for detection of puQTL. Indeed, 23.5% puQTL genes (73/310) not overlapping with eQTL genes are single-promoter genes, indicating that puQTL genes identified in this study might include false positive signals. As we mentioned above, only junction reads spanning the first and second exons were used to estimate promoter activity in this study. This reduces the number of reads that can be used to quantify, reducing the accuracy of the promoter activity estimation and the power of mapping QTL. That is a limitation of this study but we believe that our pipeline is robust when mapping QTL associated with moderately or highly expressed promoters, as we showed in some examples in the manuscript (Figure 3, 4). We now explicitly acknowledge this limitation of our analysis in the Results and Discussion sections (page 11, line number 295-303; page 18, line number 483-485).

(4) Most of the examples of novel QTLs that are identified only through puQTL methods rather than eQTL methods seem to be the result of the improved annotations conducted by the authors. This seems like a different biological message than that highlighted by the authors, which claims that mapping puQTLs itself uncovers novel regulatory genetic loci. However, instead, it seems like their pipeline does a better job annotating complete gene regions, leading to the identification of more QTL. This can be quantified by comparing how many more eQTL are identified using the improved promoter annotations. In general, it would be useful for the authors to show or speculate a bit more why they believe the puQTL associations were not detected by standard eQTL studies.

We thank the reviewer for this suggestion. In this paper, we performed eQTL mapping using the improved promoter annotations as well as puQTL mapping (Figure S2). Thus, we can claim that the improved sensitivity is due to puQTL mapping itself. We added the sentences on page 18, line number 478-480.

Minor:

(1) I know the method itself has been published elsewhere, but the authors should add a few sentences on exactly how proActiv identifies promoter activities and why these computational advances/advantages are best suited for their pipeline.

We thank the reviewer for the suggestion. We added the sentence on the page 5, line number 124-127 as follows: “The proActiv software is based on the concept of weighted splicing graphs to define unique identifiable promoters. It performs normalization of total junction reads using DESeq2, which is well used software for normalization of gene expression, and log2 transformation to obtain absolute promoter activity.”
